# Supplementary material for: Molybdenum systematics of subducted crust record reactive fluid flow from underlying slab serpentine dehydration
Source: Nat Commun. 2019 Oct 21;10:4773. doi: 10.1038/s41467-019-12696-3 (PMC6803652; doi:10.1038/s41467-019-12696-3)
Supplement: Supplementary file 1 — Supplementary Information [file 41467_2019_12696_MOESM1_ESM.pdf]

Supplementary Information for

Molybdenum systematics of subducted crust record reactive fluid flow from underlying slab serpentine dehydration

Shuo Chen et al.

Supplementary Figures

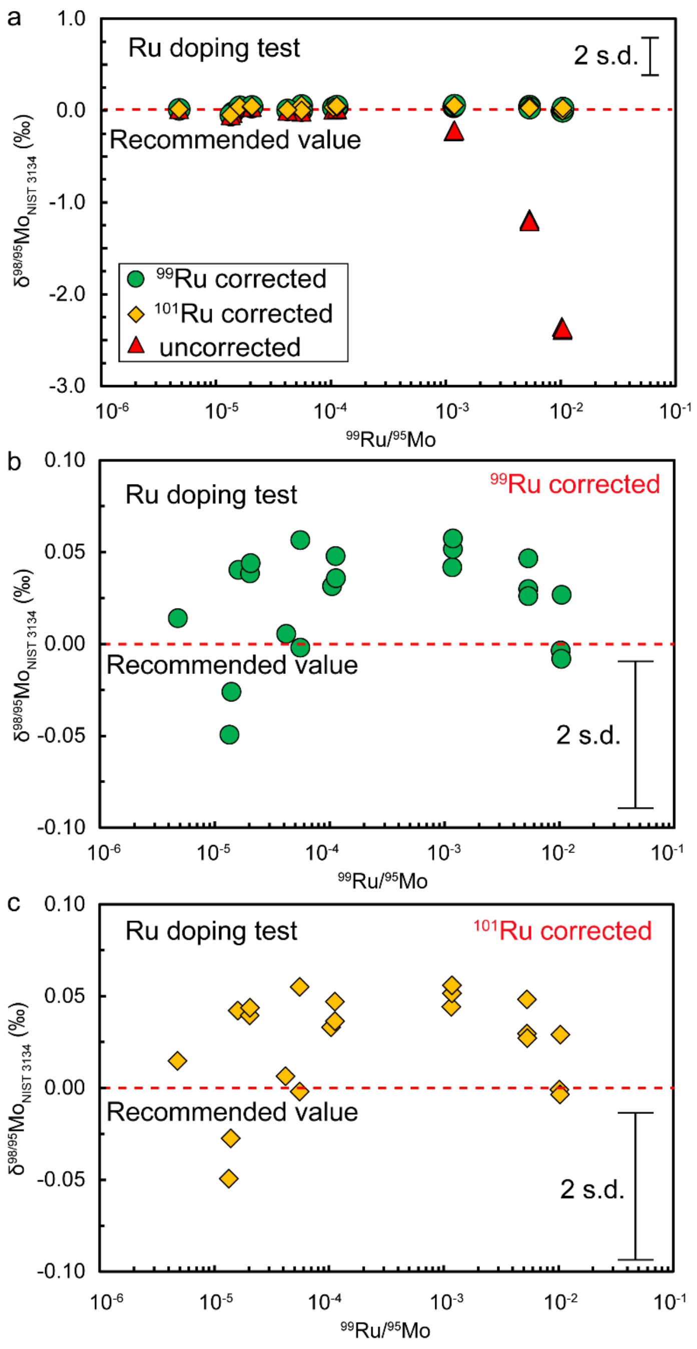

Supplementary Figure 1. Ruthenium (Ru) doping test for correction of Mo isotope data. NIST SRM 3134 was doped with different concentrations of Ru. The ^{98}Ru and ^{100}Ru interferences have been corrected using ^{99}Ru or ^{101}Ru as monitors. The effects of Ru interferences are illustrated in (a) together with close ups of corrected $\delta^{98/95}Mo_{NIST\ 3134}$ values using ^{99}Ru (b) or ^{101}Ru (c).

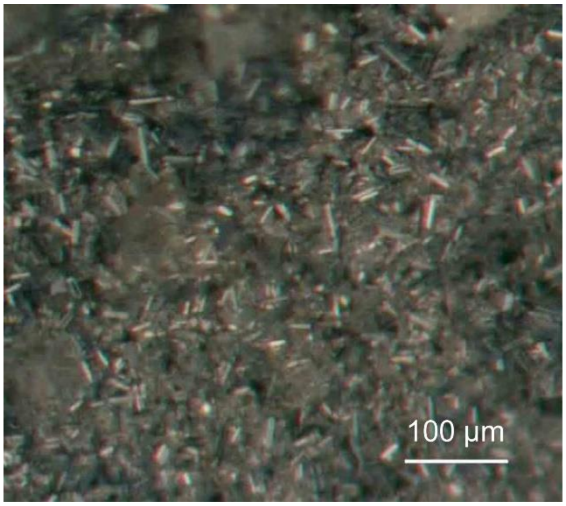

Supplementary Figure 2. Photomicrograph of undissolved residue of quenched experimental charge after 1 M HF dissolution at room temperature. Rutiles can be recognised by their lath shape.

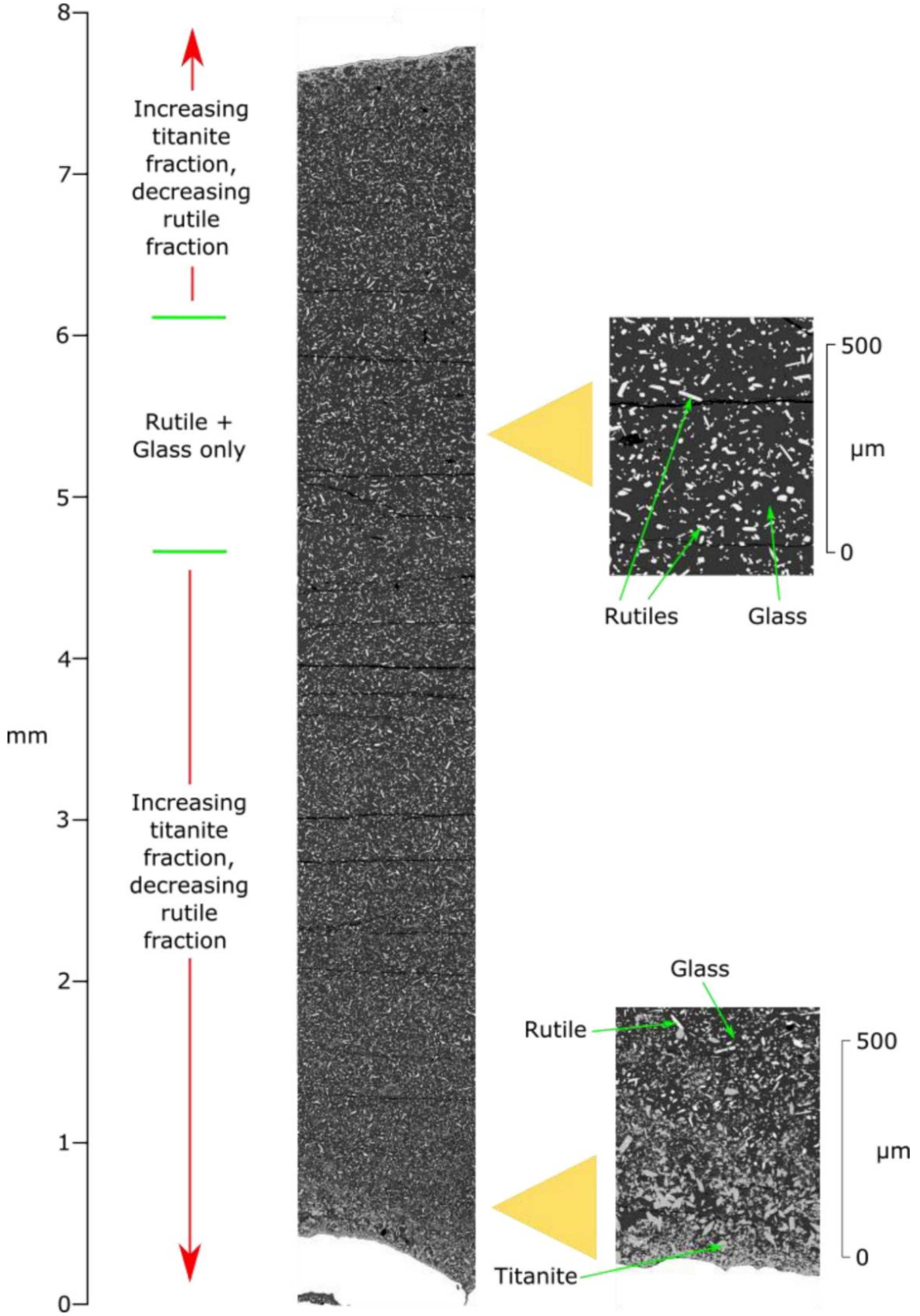

Supplementary Figure 3. Composite of back-scattered images representing the total length of the capsule. Inset images represent the titanite-free, two-phase zone of the sample as well as the titanite-richest, three-phase zone in the coldest end of the capsule.

Supplementary Tables

Supplementary Table 1. Major and trace elemental and isotopic data compilation of high-pressure mafic and ultra-mafic samples from Raspas Complex and Cabo Ortegal Complex.

**Location**	**Ecuador (Raspas Complex)**								
Sample	SEC15-2	SEC16-1	SEC42-6	SEC43-1	SEC43-3	SEC44-1	SEC46-1	SEC46-2	SEC47-1
Rock type	Blueschist	Blueschist	MORB-type eclogite	MORB-type eclogite	MORB-type eclogite	MORB-type eclogite	MORB-type eclogite	MORB-type eclogite	MORB-type eclogite
SiO_2	47.79	47.31	45.61	47.92	50.17	48.67	44.08	44.88	47.49
Al_2O_3	14.80	18.15	14.54	14.67	13.89	14.84	14.71	13.49	15.07
TiO_2	1.95	2.39	2.26	1.79	1.86	1.91	1.87	2.00	1.94
Fe_2O_3	10.65	10.18	15.14	13.04	13.30	13.75	15.48	13.77	13.87
MnO	0.20	0.16	0.28	0.19	0.23	0.19	0.24	0.21	0.23
MgO	7.00	4.44	6.16	6.46	6.65	6.00	10.09	8.72	6.01
CaO	10.50	8.36	11.84	11.10	10.67	10.84	10.87	13.41	11.15
Na_2O	3.04	3.29	3.26	3.09	3.02	3.02	1.69	2.30	2.77
K_2O	0.27	1.59	0.15	0.53	0.07	0.48	0.16	0.11	0.51
P_2O_5	0.31	0.54	0.20	0.13	0.17	0.16	0.11	0.74	0.17
LOI	3.72	2.64	0.27	0.57	0.42	0.00	0.45	0.00	0.37
Total	100.23	99.05	99.71	99.49	100.45	99.86	99.75	99.63	99.58
Li	19.40	62.50	35.20	48.50	13.80	51.40	11.00	13.30	67.30
Sc	29.00	35.60	46.00	44.30	42.60	43.20	44.80	48.00	42.90
V	209.00	254.00	415.00	376.00	390.00	389.00	362.00	431.00	358.00
Cr	265.00	24.00	187.00	214.00	111.00	211.00	253.00	252.00	256.00
Co	37.20	31.50	50.20	45.70	40.10	45.00	51.90	37.90	45.40
Ni	146.00	112.00	80.00	77.50	53.00	75.90	71.20	59.10	74.20
Cu	48.20	46.90	78.50	92.60	35.30	52.80	79.00	137.00	69.30
Zn	77.80	113.00	136.00	229.00	106.00	218.00	280.00	277.00	143.00
Ga	16.30	25.20	18.70	19.20	18.20	18.40	13.10	10.90	19.00
Rb	2.21	28.20	4.38	15.30	1.06	13.10	0.53	0.42	12.70
Sr	269.00	329.00	76.70	92.50	77.00	66.80	31.60	36.80	103.00
Ba	47.93	1466.00	77.80	98.80	21.20	79.40	6.76	21.10	60.90
Ti	11700	14340	13560	10740	11160	11460	11220	12000	11640
Y	31.90	39.30	52.60	43.10	46.10	45.10	49.10	46.40	48.00
Zr	165.00	240.00	131.00	116.00	109.00	110.00	102.00	104.00	109.00
Nb	14.80	29.20	3.64	2.04	2.40	2.03	2.19	4.26	2.09
Hf	3.82	5.23	3.68	3.01	2.85	2.89	2.86	2.91	3.13
Ta	0.93	1.73	0.24	0.13	0.14	0.13	0.14	0.18	0.15
Pb	1.28	2.44	2.97	3.81	1.50	4.06	0.65	0.57	2.90
Th	1.06	2.12	0.42	0.21	0.21	0.15	0.13	0.17	0.14
U	0.33	0.46	0.33	0.21	0.07	0.18	0.18	0.54	0.19
La	13.70	24.20	5.73	4.09	3.75	3.70	4.08	4.40	3.71
Ce	36.30	54.10	16.10	11.80	12.70	11.60	13.60	14.10	11.70
Pr	4.49	7.45	2.90	2.14	2.28	2.17	2.53	2.46	2.18
Nd	19.80	31.20	15.50	11.50	12.60	11.90	13.90	12.90	12.40
Sm	5.07	7.35	5.36	4.03	4.38	4.23	5.11	4.28	4.54
Eu	1.79	2.38	1.82	1.43	1.57	1.57	1.71	1.51	1.57
Gd	5.51	7.68	6.94	5.47	5.90	5.70	6.74	5.60	6.12
Tb	0.88	1.21	1.31	1.00	1.08	1.07	1.18	1.00	1.17
Dy	5.43	7.31	8.84	6.95	7.33	7.35	7.89	6.96	7.92
Ho	1.08	1.43	1.89	1.49	1.58	1.56	1.66	1.55	1.69
Er	2.94	3.93	5.40	4.26	4.59	4.48	4.74	4.52	4.83
Tm	0.43	0.57	0.80	0.63	0.69	0.67	0.71	0.68	0.71
Yb	2.80	3.76	5.36	4.16	4.60	4.49	4.83	4.54	4.80
Lu	0.41	0.55	0.79	0.60	0.67	0.65	0.72	0.68	0.71
$^{87}Sr/^{86}Sr$		0.704668	0.706562	0.706091	0.703781	0.706034	0.705275		0.705102
2SE		0.000005	0.000004	0.000005	0.000005	0.000005	0.000005		0.000004
$^{143}Nd/^{144}Nd$		0.512870	0.513072	0.513174	0.513214	0.513182	0.513213		0.513189
2SE		0.000003	0.000002	0.000002	0.000003	0.000003	0.000002		0.000003

Supplementary Table 1 (Continued).

**Location**	**Ecuador (Raspas Complex)**			**Cabo Ortegal (Spain)**						
Sample	SEC50-1	SEC26-3	SEC35-2	SCO1-1	SCO2-1	SCO9-2	SCO12-4	SCO16-1	SCO18-1	SCO23-1
Rock type	Retrogressed eclogite	Serpentinitized peridotite	Serpentinitized peridotite	MORB-type eclogite	MORB-type eclogite	MORB-type eclogite	MORB-type eclogite	MORB-type eclogite	MORB-type eclogite	MORB-type eclogite
SiO_2	49.57	40.24	40.49	48.72	52.33	48.42	49.62	49.22	50.21	48.45
Al_2O_3	13.62	1.62	2.37	15.73	14.15	15.22	14.27	15.04	12.72	15.97
TiO_2	1.88	0.04	0.05	1.24	1.48	1.19	1.50	1.26	2.44	1.15
Fe_2O_3	13.30	9.86	8.02	11.61	11.99	11.82	12.77	12.94	16.94	11.27
MnO	0.19	0.14	0.12	0.19	0.19	0.20	0.18	0.21	0.22	0.18
MgO	6.72	37.85	37.26	8.34	6.90	7.84	7.81	8.25	7.29	8.04
CaO	10.48	1.47	2.48	12.15	10.35	12.83	12.20	11.96	8.24	13.31
Na_2O	3.04	0.04	0.07	1.55	3.00	2.08	1.97	1.97	2.39	2.02
K_2O	0.07	0.01	0.01	0.04	0.17	0.02	0.01	0.06	0.09	0.04
P_2O_5	0.17	0.02	0.01	0.12	0.17	0.08	0.12	0.08	0.21	0.09
LOI	0.81	8.81	10.01	0.39	0.00	0.63	0.00	0.00	0.00	0.41
Total	99.85	100.10	100.89	100.08	100.73	100.33	100.45	100.99	100.75	100.93
Li	20.30	0.84	0.99	2.02	5.12	2.72	1.56	3.15	3.68	1.88
Sc	39.60	11.60	16.80	40.10	39.20	42.00	42.70	45.70	44.90	43.10
V	329.00	67.20	73.60	274.00	290.00	290.00	322.00	421.00	452.00	392.00
Cr	144.00	2758.00	2674.00	3.00	300.00	183.00	268.00	220.00	246.00	98.00
Co	38.40	126.00	104.00	44.40	39.40	47.60	44.10	48.50	46.90	41.80
Ni	47.00	2276.00	1957.00	3.00	64.00	62.00	71.00	70.00	78.00	40.00
Cu	32.90	16.20	4.82	57.00	70.60	109.20	24.50	18.80	88.60	45.50
Zn	57.40	53.00	50.30	64.60	86.70	82.80	50.10	74.90	123.60	70.50
Ga	16.30	1.52	2.10	14.10	16.30	15.80	16.60	17.20	19.10	18.00
Rb	0.54	0.03	0.22	1.02	2.17	0.04	0.43	1.30	n.d.	0.59
Sr	84.90	2.28	3.53	91.40	71.10	145.00	72.90	70.10	65.50	132.00
Ba	121.00	4.33	4.87	5.86	62.60	3.05	2.06	5.11	5.47	4.93
Ti	11280	240	300	7440	8880	7140	9000	7560	14640	6900
Y	50.50	0.78	1.58	29.90	32.70	27.80	35.80	34.30	53.40	28.70
Zr	119.00	0.16	9.49	65.30	73.70	57.90	66.20	49.80	153.00	46.10
Nb	1.91	0.01	0.01	2.68	5.27	0.96	1.78	0.96	3.70	1.48
Hf	3.25	0.01	0.24	1.79	2.11	1.68	1.98	1.56	4.01	1.40
Ta	0.14	0.00	0.00	0.16	0.33	0.06	0.11	0.06	0.25	0.09
Pb	0.74	0.28	0.28	0.34	0.70	0.71	0.28	0.67	0.37	0.70
Th	0.14	0.00	0.00	0.21	1.07	0.07	0.13	0.08	0.22	0.10
U	0.05	0.01	0.00	0.08	0.71	0.06	0.04	0.02	0.48	0.06
La	3.72	0.03	0.00	3.34	6.79	1.90	2.64	2.04	4.99	2.16
Ce	12.40	0.03	0.01	9.33	16.40	6.27	8.56	6.25	15.80	7.07
Pr	2.32	0.01	0.01	1.57	2.43	1.19	1.57	1.30	2.75	1.30
Nd	12.70	0.05	0.07	8.43	12.00	6.94	8.91	7.51	15.20	7.37
Sm	4.62	0.03	0.07	2.91	3.82	2.64	3.38	2.83	5.32	2.79
Eu	1.59	0.01	0.03	1.03	1.28	1.00	1.23	1.03	1.87	1.07
Gd	6.34	0.07	0.15	3.90	4.59	3.62	4.55	3.93	7.20	3.82
Tb	1.17	0.02	0.03	0.72	0.79	0.69	0.86	0.77	1.35	0.73
Dy	8.01	0.12	0.27	4.83	5.45	4.70	5.87	5.43	9.05	4.88
Ho	1.71	0.03	0.06	1.06	1.21	1.03	1.28	1.19	1.93	1.02
Er	4.89	0.09	0.19	2.97	3.45	2.91	3.67	3.42	5.41	2.92
Tm	0.73	0.02	0.03	0.44	0.52	0.44	0.54	0.50	0.81	0.43
Yb	4.89	0.12	0.20	2.91	3.47	2.92	3.61	3.31	5.43	2.85
Lu	0.73	0.02	0.03	0.43	0.51	0.43	0.52	0.50	0.80	0.42

Data soureces: Raspas Complex: Petrology and major, trace elements are from Ref.[1], and Sr-Nd isotopes are from Ref.[2]; Cabo Ortegal Complex: Major, trace elements are from Ref.[3].

Supplementary Table 2. Compositions of melt, rutile and titanite in the experimental run product.

	**Melt**	**Rutile**	**Titanite**
SiO_2	53.3 (0.4)	0.2 (0.2)	30.1 (0.4)
Al_2O_3	19.4 (0.2)	1.3 (0.1)	1.9 (0.2)
CaO	6.9 (0.2)	0.26 (0.03)	28.8 (0.3)
TiO_2	5.8 (0.2)	94.7 (0.4)	38.6 (1.0)
Na_2O	3.9 (0.4)	0.01 (0.07)	0.04 (0.05)
K_2O	7.5 (0.2)	0.19 (0.03)	0.13 (0.03)
H_2O*	4.4 (0.4)	n.a.	n.a.
Mo	472 (51)	360 (29)	364 (177)
$\delta^{98/95}Mo_{NIST\ 3134}$ (‰)	0.02 (0.05)	-0.28(0.01)	
$\delta^{98/95}Mo_{NIST\ 3134}$ (‰)	0.04(0.01)	-0.32(0.01)	

* Water contents calculated by difference to 100%. All oxides in weight percent, Mo in µg/g. Note that Ti was calibrated on an ilmenite crystal, probably causing the poor total for rutile. Mo isotope compositions are given for both digestions of glass as well as rutile. Values in brackets are standard deviations, n.a. = not appropriate.

Supplementary Table 3. $\delta^{98/95}Mo_{NIST\ 3134}$ and Mo concentration of repeated digestions of W-2a (n = 13), JB-2 (n = 5), and AGV-2 (n = 5) in this study. Each digestion was measured several times.

Sample	$\delta^{98/95}Mo_{NIST\ -3134}$ (‰)	2SD	[Mo] (μg g^{-1})	n	Digestion method	Sample	$\delta^{98/95}Mo_{NIST\ -3134}$ (‰)	2SD	[Mo] (μg g^{-1})	n	Digestion method
W-2a (01)	-0.02	0.03	0.40	3	Bomb	JB-2(1)	0.04	0.02	0.87	2	Hotplate
W-2a (01)	-0.01	0.04	0.40	3	Bomb	JB-2(2)	0.00	0.03	1.05	2	Hotplate
W-2a (02)	-0.07	0.03	0.40	3	Bomb	JB-2(3)	0.03	0.01	0.94	2	Hotplate
W-2a (02)	-0.02	0.00	0.40	2	Bomb	JB-2(3)	0.05	0.06	0.93	3	Hotplate
W-2a (03)	-0.01	0.02	0.44	3	Bomb	JB-2(4)	0.04	0.01	1.05	2	Hotplate
W-2a (04)	-0.04	0.08	0.43	3	Bomb	JB-2(5)	0.03	0.03	0.91	3	Hotplate
W-2a (04)	-0.03	0.09	0.43	3	Bomb	Mean	0.03		0.96		
W-2a (05)	-0.05	0.01	0.42	3	Bomb	2SD	0.03		0.15		
W-2a (05)	-0.06		0.42	1	Bomb	AGV-2(1)	-0.17	0.1	1.91	2	Bomb
W-2a (06)	-0.06	0.03	0.43	3	Bomb	AGV-2(2)	-0.16	0.06	1.90	2	Hotplate
W-2a (07)	-0.03	0.09	0.40	3	Hotplate	AGV-2(3)	-0.15		1.85	1	Bomb
W-2a (07)	-0.02	0.01	0.4	3	Hotplate	AGV-2(3)	-0.15	0.06	1.84	2	Bomb
W-2a (08)	-0.02	0.05	0.41	2	Hotplate	AGV-2 (3)	-0.13	0.05	1.84	3	Bomb
W-2a (09)	-0.01	0.05	0.40	3	Hotplate	AGV-2(4)	-0.20		1.89	1	Bomb
W-2a (09)	0.00	0.09	0.40	3	Bomb	AGV-2(4)	-0.16	0.02	1.89	2	Bomb
W-2 (10)	-0.02	0.05	0.43	3	Bomb	AGV-2(4)	-0.18	0.03	1.89	3	Bomb
W-2 (10)	-0.03	0.03	0.43	3	Bomb	AGV-2(5)	-0.14	0.05	1.87	3	Hotplate
W-2a (11)	-0.02	0.05	0.41	3	Bomb	Mean	-0.16		1.88		
W-2a (11)	-0.02	0.02	0.41	3	Bomb	2SD	0.04		0.05		
W-2a (11)	-0.02	0.04	0.41	3	Bomb						
W-2a (11)	-0.05	0.01	0.41	3	Bomb						
W-2a (11)	-0.03	0.03	0.41	3	Bomb						
W-2a (11)	-0.01	0.03	0.41	3	Bomb						
W-2a (12)	-0.04	0.03	0.39	3	Bomb						
W-2a (12)	-0.05	0.02	0.39	3	Bomb						
W-2a (13)	-0.07	0.05	0.42	3	Bomb						
W-2a (13)	-0.05	0.02	0.42	3	Bomb						
Mean	-0.03		0.41								
2SD.	0.04		0.03								

Hotplate refers to samples digested by hotplate. Bomb refers to samples digested by high pressure bomb.

Supplementary Table 4. Mineral/fluid partition coefficients of Mo and Ce at 2.6 GPa, 600 °C and parameters used for calculating residual eclogite compositions in Fig. 2.

**Mineral/fluid partition coefficients**	**Mo§**	**Ce‡**
$D^{clinopyroxene/fluid}$	$[40/10^{(0.44\times \log fO2+0.42\times \log NaCl-1.8\times 1000/T+4.8)}]\times \varphi_{600°C}$	2
$D^{garnet/fluid}$	$[12/10^{(0.44\times \log fO2+0.42\times \log NaCl-1.8\times 1000/T+4.8)}]\times \varphi_{600°C}$	0.4
$D^{rutile/fluid}$	$[87670/10^{(0.44\times \log fO2+0.42\times \log NaCl-1.8\times 1000/T+4.8)}]\times \varphi_{600°C}$	2
Mineral assemblage	Cpx (68.9%) + Grt (29.6%)+Rt (1.5%)	
Parameters used in the models.	Depleted MORB (Average)	Ref.
$\delta^{98/95}Mo_{NIST\ 3134}$ (‰)	-0.21	4
Mo (μg g^{-1})	0.19	4
Ce (μg g^{-1})	5.6	4

§ Mineral/fluid partition coefficients of Mo was calculated at 600 °C based on ref.[5], where $\varphi_{600°C}$ denotes correcting factor of temperature dependence of Zr in rutile (ref.[6]) from 1000 °C to 600 °C, e.g., $\varphi_{600°C}$=[Zr-in-rutile$_{600°C}$]/[Zr-in-rutile$_{1000°C}$]=0.023; ‡ Mineral/fluid partition coefficients of Ce are from ref.[5] and references therein.

Supplementary References

1. John T, Scherer EE, Schenk V, Herms P, Halama R & Garbe-Schönberg D. Subducted seamounts in an eclogite-facies ophiolite sequence: the Andean Raspas Complex, SW Ecuador. *Contrib. Mineral. Petrol.* **159**, 265-284 (2010).
2. Halama R, John T, Herms P, Hauff F & Schenk V. A stable (Li, O) and radiogenic (Sr, Nd) isotope perspective on metasomatic processes in a subducting slab. Chem. Geol. 281, 151-166 (2011).
3. Halama R, Bebout GE, John T & Schenk V. Nitrogen recycling in subducted oceanic lithosphere: The record in high- and ultrahigh-pressure metabasaltic rocks. *Geochim. Cosmochim. Acta* **74**, 1636-1652 (2010).
4. Bezard R, Fischer-Gödde M, Hamelin C, Brennecka GA & Kleine T. The effects of magmatic processes and crustal recycling on the molybdenum stable isotopic composition of Mid-Ocean Ridge Basalts. *Earth Planet. Sci. Lett.* **453**, 171-181 (2016).
5. Bali E, Keppler H & Audetat A. The mobility of W and Mo in subduction zone fluids and the Mo–W–Th–U systematics of island arc magmas. *Earth Planet. Sci. Lett.* **351-352**, 195-207 (2012).
6. Schmidt A, Weyer S, John T & Brey GP. HFSE systematics of rutile-bearing eclogites: New insights into subduction zone processes and implications for the earth's HFSE budget. *Geochim. Cosmochim. Acta* **73**, 455-468 (2009).
